# Divergent Allometric Trajectories in Gene Expression and Coexpression Produce Species Differences in Sympatrically Speciating Midas Cichlid Fish

**DOI:** 10.1093/gbe/evz108

**Published:** 2019-05-24

**Authors:** Carmelo Fruciano, Axel Meyer, Paolo Franchini

**Affiliations:** 1 Department of Biology, University of Konstanz, Germany; 2 Institut de Biologie de l'École Normale Supérieure (IBENS), École Normale Supérieure, CNRS UMR 8197, Paris, France

**Keywords:** RNA-Seq, coexpression, WGCNA, benthic–limnetic divergence, evolvability, modules

## Abstract

The mechanisms of speciation without geographic isolation (i.e., sympatric speciation) remain debated. This is due in part to the fact that the genomic landscape that could promote or hinder species divergence in the presence of gene flow is still largely unknown. However, intensive research is now centered on understanding the genetic architecture of adaptive traits associated with this process as well as how gene expression might affect these traits. Here, using RNA-Seq data, we investigated gene expression of sympatrically speciating benthic and limnetic Neotropical cichlid fishes at two developmental stages. First, we identified groups of coexpressed genes (modules) at each stage. Although there are a few large and well-preserved modules, most of the other modules are not preserved across life stages. Second, we show that later in development more and larger coexpression modules are associated with divergence between benthic and limnetic fish compared with the earlier life stage. This divergence between benthic and limnetic fish in coexpression mirrors divergence in overall expression between benthic and limnetic fish, which is more pronounced later in life. Our results reveal that already at 1-day posthatch benthic and limnetic fish diverge in (co)expression, and that this divergence becomes more substantial when fish are free-swimming but still unlikely to have divergent swimming and feeding habits. More importantly, our study describes how the coexpression of several genes through development, as opposed to individual genes, is associated with benthic–limnetic species differences, and how two morphogenetic trajectories diverge as fish grow older.

## Introduction

Understanding complex evolutionary processes benefits from an integrative approach across levels of biological organization. This is the case, for example, for sympatric speciation. Theoretical models suggest that speciation in the face of gene flow is possible (Via 2001; Gavrilets 2004; Bolnick and Fitzpatrick 2007), and it might be a more common phenomenon than previously thought (Savolainen et al. 2006; Papadopulos et al. 2014). However, relatively few widely accepted empirical cases of sympatric speciation have been documented so far (reviewed in Bolnick and Fitzpatrick 2007) and it is still not clear what genomic mechanisms or preconditions accompany or facilitate this process. In the past decade, thanks to the emergence of new, powerful and financially more accessible sequencing technologies, a growing body of research has been dedicated to understanding the molecular genetic mechanisms that could promote or hinder speciation-with-gene-flow (Feder and Nosil 2010). For example, it is now possible to more deeply investigate how the genomic architecture of adaptive traits can influence speciation-with-gene-flow (Nosil and Feder 2012; Flaxman et al. 2014; Fruciano et al. 2016a; Wolf and Ellegren 2017), and how differential gene expression is associated with variation in these traits (Pavey et al. 2010). These accumulating genomic data, integrated in a multidisciplinary approach involving biotic and environmental parameters, are opening new exciting perspectives for discovering the conditions underlying sympatric speciation. 

**Fig. 1.**
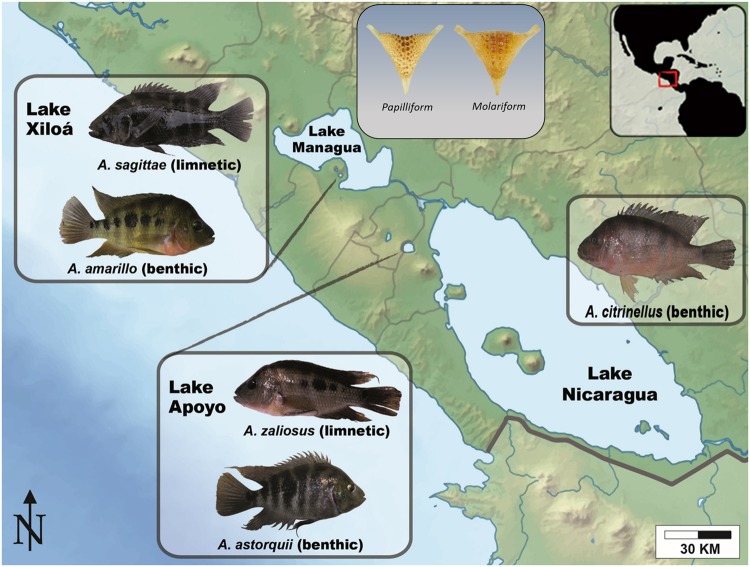
—Map of the Nicaraguan lake system highlighting the two largest lakes (Managua and Nicaragua) and the two crater lakes housing benthic–limnetic species pairs (Apoyo and Xiloá). In the insets, pictures of representative specimens of the benthic species *Amphilophus citrinellus* from Lake Nicaragua, and the benthic (*A. astorquii* and *A. Amarillo*) and limnetic (*A. sagittae* and *A. zaliosus*) species from the crater lakes are shown. Benthic species tend to have deeper, more robust pharyngeal jaw than their limnetic counterparts (called “molariform” and “papilliform,” respectively; representative pictures shown in the upper inset). The figure (background map and insets) was adapted from previous versions (Franchini et al. 2014a, 2016b; Fruciano et al. 2016a) published under a Creative Commons (CC) license.

The *Amphilophus citrinellus* species complex from Nicaragua, fish known as Midas cichlids, represents one of the few widely accepted cases of sympatric speciation. This group of Neotropical cichlids, through small parallel adaptive radiations, have repeatedly diverged into bottom-dwelling (deeper-bodied benthic) and open-water (elongated limnetic) species from a common benthic ancestor in at least two young crater lakes (fig. 1). Midas cichlids have been the subject of intensive research, which has investigated different aspects of their biology in an effort to better understand the processes promoting sympatric speciation (with repeatedly evolved parallel phenotypic outcomes) (Barluenga and Meyer 2004; Elmer et al. 2009, 2010b; Muschick et al. 2011). For instance, some studies have investigated the morphological traits that underlie divergence in sympatry by testing in which way these traits diverge, whether this divergence is the same across different crater lakes, and whether the divergence observed in nature is maintained in fish kept under the same laboratory conditions (Barluenga et al. 2006; Elmer et al. 2010b; Franchini et al. 2014b; Fruciano et al. 2016a). Other studies have investigated the level of genetic divergence in natural populations of these species to understand the level of gene flow, as well as the timing and order of divergence events in this species (Barluenga and Meyer 2010; Elmer et al. 2010b; Kautt et al. 2016).

Recently, it has also been shown that, in agreement with theoretical models of divergence (Flaxman et al. 2014), the genetic nonindependence of different adaptive traits may be one of the factors facilitating the speciation process in the presence of gene flow (Fruciano et al. 2016a). Quantitative trait loci (QTL) mapping studies (Franchini et al. 2014b; Fruciano et al. 2016a) can help us understand how the broad-scale genetic architecture of adaptive traits can promote sympatric divergence. However, they cannot elucidate whether the very recent and explosive diversification of these fish is sustained by mutations in protein coding regions or, rather, by variation in regulatory regions (Elmer and Meyer 2011). A large number of studies have found evidence for the association between gene regulatory processes and phenotypic variation (Krubitzer and Kaas 2005; Romero et al. 2012; Salinas et al. 2016), and have stressed the crucial role of gene regulation in early adaptive divergence (Wittkopp et al. 2003; Chan et al. 2010; Romero et al. 2012; Seehausen et al. 2014). For example, it has been recently shown how evolutionary divergence at the 3’ UTRs (three prime untranslated regions), regions known to have an important gene regulatory role in a plethora of pivotal biological processes, could have contributed to the rapid diversification of cichlid fishes (Xiong et al. 2018). More specifically, recent evidence based on the analysis of population-level genomic data at putative microRNA binding sites (Franchini et al. 2016b) suggests that divergence in regulatory regions might be one of the main factors underlying sympatric divergence, also in Midas cichlids. However, previous transcriptomic studies in cichlids mainly investigated the possibility that divergence in sympatry is due to nonneutral sequence evolution at protein coding genes (Elmer et al. 2010a; Fruciano et al. 2016a). Perhaps more importantly, previous studies targeting the coding part of the genome—in these fish and more generally in evolutionary studies—have usually adopted a single-gene approach. Although this strategy is useful as it reduces the complexity of gene expression patterns, at the same time it disregards patterns of covariance among genes, thus leading to loss of important and evolutionarily meaningful information. This is particularly true in the cases—such as with Midas cichlids—where divergence between two or more forms occurs in complex (i.e., polygenic) traits, where one can safely assume pleiotropy, and concerted, interacting variation of the expression of many genes in complex interaction networks.

In the present study, we focus mainly on pattern of coexpression between genes. Particularly, we investigate whether any patterns of expression and coexpression, divergent between benthic and limnetic species of Midas cichlids, are the same and involve the same genes across different life stages. While most studies usually focus on analyzing adults (Elmer et al. 2010a; Henning et al. 2013; Manousaki et al. 2013), here we focus on two early life stages: 1 day posthatching (1 dph) and 1 month posthatching (1 mph). One of the reasons we focused on nonsexually mature fish is that already at young age (a few centimeters of standard length) differences between benthic and limnetic fish in external morphology (body shape) can already be noticed by the naked eye (C.F., personal observation). The two stages we focus on are sufficiently distinct to ask whether there are “allometric trajectories” in gene (co)expression. In fact, at 1 dph Midas cichlid fish are larvae attached to the substrate and have not yet started to eat autonomously (they are consuming the yolk sac). Instead, at 1 mph Midas cichlids are already swimming freely and nearly have already the morphology of adult fish. Therefore, this experimental design in this study system also allows us to test two alternative hypotheses with regard to divergence in patterns of (co)expression between benthic and limnetic fish across life stages. Provided that we can safely assume that patterns of gene (co)expression will change over time during development (e.g., Song et al. 2015) and are therefore “allometric,” it might be the case that the difference between benthic and limnetic fish becomes larger as the fish grow older. This hypothesis therefore posits divergent “allometric trajectories” in (co)expression. This hypothesis naturally follows from the observation that the main differences this far documented between benthic and limnetic Midas cichlids are in swimming-related external morphology (i.e., body shape) and trophic morphology (i.e., pharyngeal jaw size and shape) (Meyer 1989, 1990a, 1990b; Franchini et al. 2014b; Fruciano et al. 2016a). For this reason, it seems reasonable to presume that the patterns of gene (co)expression are different between larvae which do not eat nor swim and fully swimming and eating fish, but also that the divergence between benthic and limnetic fish will be larger at the stage when they do swim and eat autonomously (1 mph). However, an alternative—perhaps less likely—hypothesis is that the divergence between benthic and limnetic fish is already substantial at 1 dph and similar in extent to the divergence in (co)expression observed at 1 mph. In other words, the two “allometric trajectories” of (co)expression in benthic and limnetic fish would be roughly parallel. This hypothesis follows from the idea that the differences in morphology observed at the later stage should be preceded by variation in gene expression causing the variation in morphology (e.g., Schneider et al. 2014), therefore the divergence in gene expression at an earlier age could potentially be as large (or even larger) than the one observed later (Aubin-Horth and Renn 2009). This hypothesis becomes even more realistic if one considers that, while the more clearly documented differences between benthic and limnetic Midas cichlids are in external and trophic morphology (but see Franchini et al. 2014a), differences related to swimming mode and trophic habit can also be physiological (e.g., ability to sustain prolonged swimming in open water) and could occur earlier during development than the morphological differences documented this far.

## Materials and Methods

### Data Sampling

For this study, we used five cichlid species belonging to the Midas group: Two benthic/limnetic species pairs, one from crater Lake Apoyo (*Amphilophus astorquii* and *A. zaliosus*) and one from crater Lake Xiloá (*A. amarillo* and *A. sagittae*), and a benthic species from Lake Nicaragua (*A. citrinellus*) (fig. 1). These fishes are derived from fish caught in 2007 in Lake Apoyo, Lake Xiloá and Lake Nicaragua (Elmer et al. 2010a), with the permission of MARENA (Ministry of the Environment and Natural Resources). For each species, the adults have been laboratory-reared under common conditions at the University of Konstanz animal facility (TFA) since the time of collection. After 3–4 years, broods were raised and sampled at two developmental stages, 1 dph and 1 mph. Due to the small amount of RNA obtained from 1 dph embryos, three individuals were pooled in one sample for downstream sequencing library construction (15 libraries in total). For the 1 mph stage, to maximize the number of unique transcripts, from a single fish, bodies and heads were separated and treated as different samples (for a total of 24 libraries) (see supplementary table S1, Supplementary Material online for the details). For this experiment, we used whole organism-derived expression data. As we were mainly interested in the association between coexpression modules and benthic–limnetic divergence, and we are aware of the (highly) polygenic basis of this complex phenotype, we believe that our experimental design allowed us to capture general patterns of gene expression and coexpression between developmental stages and species. This study used previously published data; therefore, specific ethics approval was not required for the current study. Our previous studies using these data (Franchini et al. 2016b; Fruciano et al. 2016a) were authorized by ethical permits by the Regierungspräsdium Freiburg, Abteilung Landwirtschaft, Ländlicher Raum, Veterinär- und Lebensmittelwesen.

### Library Construction and Sequencing

A FastPrep-24 homogenizer (MP Biomedicals, Santa Ana, USA) was used to process 30 μg of each sample (30 s at 4.0 M), following isolation of total RNA using a Qiagen RNeasy Mini Kit (Qiagen, Valencia, USA). A Qubit v2.0 fluorometer (Life Technologies, Darmstadt, Germany) and a Bioanalyzer 2100 (Agilent Technologies, Palo Alto, USA) were used to assess RNA quantity and quality, respectively. Four-hundred ng of high-quality RNA (RIN value >8) was used to construct barcoded RNA sequencing (RNA-Seq) libraries with the Illumina TruSeq RNA sample preparation kit v2 (Low-Throughput protocol) according to the manufacturer’s instructions (Illumina, San Diego, USA). A total of 39 libraries were paired-end sequenced on an Illumina HiSeq2500 (2× 151 bp) at the genome facility at the University of Tufts (TUCF Genomics, Boston, USA).

### Transcriptome Assembly and Gene Expression

To eliminate the remaining adapters and to quality filter the raw sequences, we used the program Trimmomatic v0.33 (Bolger et al. 2014) in default mode, discarding sequences shorter than 50 bp. Filtered reads of the 39 samples were combined and assembled using a reference-guided approach. Briefly, reads were aligned to the Midas genome v7.5 (unpublished version) using the splicing-aware mapping program Hisat2 v2.1.0 (Kim et al. 2015) in default mode. The mapping output, converted from SAM to BAM and sorted, was then processed by Stringtie v1.3.3b (Pertea et al. 2016) to assemble RNA-Seq alignments into potential transcripts. Transcripts were then extracted from the Midas genome using the *gffread* utility implemented in the Cufflink v2.2.1 package (Trapnell et al. 2010) and used as query in a BLASTx search (v2.2.26 algorithm) against the Nile tilapia, *Oreochromis niloticus*, protein data set (source Ensembl release 90) enforcing a cutoff e-value of 1e^−6^. The longest transcripts among those matching the same tilapia protein was retained.

The obtained final set of sequences was further clustered using the program Corset v1.06 (Davidson and Oshlack 2014). First, read mapping for each sample was performed independently (bodies and heads read sets for each replicate were merged to form a single sample/replicate) using Bowtie v2.2.3 (Langmead and Salzberg 2012) allowing infinite number of alignments (-a). Second, the alignment files were processed by Corset that identified the minimum number of clusters in the transcript data set (where each “cluster” represents a gene) and at the same time created the raw expression table for all the given samples. The log-likelihood ratio (-D) Corset parameter was set to 20,000 and only transcripts with a minimum of 20 aligned reads were retained for downstream analysis.

### Read Count Preprocessing

To ensure that our analysis was based on reliable read count data, we excluded from the analysis those transcripts with less than 100 reads across all samples and less than 50 reads across all individuals of each growth stage. This filtering step left with a total of 69.2 M reads for 1 dph (mean per library 4.6 M; standard deviation 1.8 M) and 78.2 M reads for 1 mph (mean per library 6.5 M; standard deviation 1.4 M). The subsequent preprocessing steps were applied to each stage separately and generally following the suggestions of the WGCNA (Weighted Correlation Network Analysis) package user guide and accompanying book (Horvath 2011) (details and departures from default choices will be provided below). Data quality was further checked in the R package WGCNA v1.63 (Langfelder and Horvath 2008) for missing entries and zero-variance transcripts and each data set was then subjected to the variance stabilizing transformation implemented in the R statistical package DESeq2 v1.22.2 (Love et al. 2014). Furthermore, an iterative procedure of identification and removal of outliers was applied based on standardized connectivity (Horvath 2011) using as threshold value −2 to identify outliers, removing observation identified as outliers and then repeating the computation of the adjacency network, repeating the procedure until no observations were deemed outliers. In this outlier removal step, an *A. amarillo* 1 dph, an *A. zaliosus* 1 dph, and an *A. astorquii* 1 mph were removed from the analysis. Finally, the typical WGCNA procedure for the choice of the soft thresholding power was followed (with powers from 1 to 30) and a soft thresholding power of 20 was chosen as this value is the one recommended when the threshold of 0.9 for the scale free topology criterion is not reached (which was our case) (a flowchart describing the main WGCNA-related analyses is provided as supplementary figure S2, Supplementary Material online).

### Gene Coexpression within Stages

Automatic module detection in WGCNA was used on each growth stage (1 dph and 1 mph) to identify modules of coexpressed transcripts based on a signed network computed using biweight midcorrelation. The analysis was performed to give a single network with all the transcripts and using as minimum module size (number of transcripts in a module) 30, 0.1 as maximum percentile for outliers and 0.25 as dendrogram cut height.

For each module of coexpressed genes thus obtained, we computed “eigengenes” (Langfelder and Horvath 2007). These are not real biological entities (i.e., genes or transcripts) but, rather, a statistical construct useful to summarize a module, obtained as the first principal component of the expression matrix of each module. The advantage is that this statistical summary can be used to identify, for instance, modules associated with biological properties (Langfelder and Horvath 2007).

In our case, indeed, we used “eigengenes” as a tool to identify modules of coexpressed genes potentially associated with the benthic/limnetic state by computing the biweight midcorrelation of “eigengenes” (i.e., individual scores on each “eigengene”) and benthic/limnetic state (avoiding the robust estimation of this as it is inappropriate for binary predictors) and assessing its significance. To avoid false positives and reduce the number of modules to a small number of modules more robustly associated with benthic/limnetic state, we applied the Benjamini–Hochberg procedure to control false discovery rate (Benjamini and Hochberg 1995). To further assess the validity of the modules identified in this way, we performed two permutation tests. In the first, we tested the variance explained by the first eigenvalue. This test follows the same principles as permutation testing used in parallel analysis to determine the number of principal components to retain (Buja and Eyuboglu 1992). In particular, here we randomly permuted rows (observations) so that the association between different variables (gene expression of each gene) is disrupted. This gives a null distribution of eigenvalues against which the observed eigenvalue can be compared. This procedure allows computing a *P*-value (as the proportion of eigenvalues obtained under the null larger or equal to the observed eigenvalue) to determine whether a given principal component has a sufficiently large eigenvalue compared with “noise.” The variance explained by each eigenvalue equals the eigenvalue divided the sum of eigenvalues and here only the first eigenvalue is of interest (because only the first principal component is retained as “eigengene”). For these reasons, here we tested only for the first eigenvalue of the selected modules using explained variance as test statistic. We performed this analysis using both the correlation coefficient and biweight midcorrelation using 1,000 random permutations. In the context of the present study, this test has the function of excluding the possibility that the “eigengene” identified merely represents noise.

For the second test of the modules identified, we focused on the multivariate association between gene expression in these modules and benthic/limnetic state. The reasoning behind this test is that the correlation between “module eigengene” and benthic/limnetic state performed above (and in the literature using WGCNA) reduces multivariate data (gene expression across genes in the module) to its first principal component (i.e., a univariate projection). This, for instance, could lead to overemphasizing the association between module gene expression and phenotype (i.e., benthic/limnetic state). To address this potential limitation, here we used a multivariate analog of the correlation coefficient, Escoufier RV (Escoufier 1973), to test for the significance of the association between module gene expression and benthic/limnetic state. While this coefficient is largely unused in the analysis of genomic and transcriptomic data and its value cannot be interpreted directly because it depends on sample size and number of variables (Smilde et al. 2009; Fruciano et al. 2013), it is widely used in evolutionary biology and other fields to test for multivariate association through permutation (e.g., Génard et al. 1994; Klingenberg 2009; Fruciano et al. 2013, 2016b; Josse and Holmes 2016; Chiozzi et al. 2018). In this study, we performed the test by randomly permuting (100 permutations) the benthic/limnetic labels while maintaining the matrix of gene expression for the module under consideration and computing Escoufier RV at each permutation. This disrupts the association between module gene expression and benthic/limnetic state, so that the empirical distribution of the Escoufier RV obtained through permutations reflects the null hypothesis of no association between module expression and benthic/limnetic state. A *P*-value is then computed as the proportion of permuted RV values larger or equal to the one observed.

To facilitate their future use by other researchers, we distribute the R implementation of the two permutation tests described above in the package *resampleWGCNA* (available at https://github.com/fruciano/resampleWGCNA). The modules thus selected obviously contain genes with different levels of association with benthic/limnetic state and with different levels of reliability in the assignment to a given module. For these reasons, to derive a robust set of candidate transcripts, for each of the selected modules, we obtained the transcripts with absolute value of both module membership (correlation of expression with “eigengene” scores) and biweighted midcorrelation with benthic/limnetic state higher than 0.7. We consider these a strong set of candidate transcript coexpressing in expression modules, confidently assigned to a given module and associated to benthic/limnetic state. 

### Analyses of Differential Gene Expression

While our study focuses on coexpression and global patterns of gene expression, we also performed a more traditional gene-by-gene analysis of differential gene expression to confirm our results. For this analysis, we used the program DESeq2 (Love et al. 2014) and tested—separately for 1 dph and 1 mph—for differential expression between benthic and limnetic fish. False discovery rate in the results of the DESeq2 analyses was controlled at the 0.05 level using the Benjamini–Hochberg procedure. To test whether the modules we identified as robustly associated with benthic/limnetic state were enriched with differentially expressed genes compared with the rest of the transcriptome, we performed a series of Fisher’s exact tests. In this way, we tested for the frequency of genes significantly and nonsignificantly differentially expressed in each of the selected modules and their frequency outside of any selected module.

### Overlap of Selected Modules and Previously Identified QTL

We also quantified the level of physical overlap between the modules we identified here as robustly associated with benthic/limnetic state and the QTL regions for adaptive traits we identified in a previous study (Fruciano et al. 2016a). There, using a combination of RAD-Seq, advanced morphometrics and multivariate QTL mapping, we identified a number of QTL regions for body shape and lower pharyngeal jaw shape, as well as a region where the QTL for these two traits overlapped and where we found a “QTL for covariation” between the two traits. Here, we identified these QTL regions in the Midas genome v7.5 (the QTL study used a previous version of the genome) by blasting the RAD markers in the QTL regions on the new version of the Midas genome. In the same way, we also identified the position of each of the transcripts in the three coexpression modules associated with benthic/limnetic state (royal blue 1 dph, turquoise 1 mph, black 1 mph). Finally, we tested for significance of the number of overlaps between the genes in each module and QTL regions (all together and separately) using the permutational procedure implemented in *regioneR* (Gel et al. 2016). This procedure allows for testing the significance of overlaps between genomic regions identified a priori, while accounting for the size of the genome, as well as its arrangement in linkage groups and chromosomes. This procedure is particularly useful in the present study because, obviously, modules with more genes will have higher chances of being found in QTL regions and, at the same time, larger QTL regions will be more likely to contain genes in the selected modules. To further ensure robustness of this procedure, we restricted it to genomic regions assigned to linkage groups in the Midas genome, avoiding unplaced scaffolds. To reduce false positives due to the potentially large number of tests, we also controlled for false discovery rate by using the Benjamini–Hochberg procedure. Furthermore, we repeated this procedure also for all the genes deemed significant by the DESeq2-based gene-by-gene analysis of differential gene expression separated by stage, as well as the genes which were at the same time differentially expressed between benthic and limnetic fish and in one of the selected modules.

### Exploratory Analyses of Global Transcription Profiles

To explore whether any difference across life stages in association between coexpression patterns and benthic/limnetic state could be due to “allometric trajectories” in gene expression, we performed two additional exploratory analyses. Starting from the original data (prior to removal of low-count transcripts and outlier specimens), we applied a common variance stabilizing transformation in DESeq2 (so that transcript abundances would be comparable across stages). Then, we used unique combinations of life stage and benthic/limnetic state (i.e., 1 dph benthic, 1 dph limnetic, 1 mph benthic, 1 mph limnetic) as grouping factor and computed Euclidean distances between groups to verify whether the distance between benthic and limnetic overall expression patterns was similar across life stages. We also used the same groupings to perform a between-group principal component analysis (Boulesteix 2005). This is an ordination technique that has gained popularity in other biological areas, such as morphometrics, due to its ability to visualize variation among groups and its advantages relative to other ordination techniques such as canonical variate analysis (Mitteroecker and Bookstein 2011; Franchini et al. 2014b, 2016a). For the present study, to overcome the necessity of computing an extremely high-dimensional covariance matrix, we first computed Euclidean distances among individual observations, then performed a principal coordinates analysis of these distances, and finally performed on the principal coordinates scores thus obtained the between-group principal component analysis itself. These computations and visualizations employed the R packages *ape* v5.1 (Paradis et al. 2004), *ggplot2* v3.1.1 (Wickham 2009), and *Morpho* v2.6 (Schlager 2017).

### Patterns of Gene Coexpression across Stages

To study the level of preservation of coexpression patterns across stages, we performed an analysis of module preservation, as implemented in WGCNA, using the 1 mph stage network as reference and the 1-dph stage network as test. This analysis quantifies and tests (here, using 1,000 permutations) how well modules in the reference network are maintained in the test network. Here, we use the statistic *Z*_summary_ (Langfelder et al. 2011) to quantify the degree of preservation of the 1 mph modules in the 1-dph stage. This statistic is a composite measure of the various analyses performed by the module preservation function of WGCNA. After removal of a few genes using the *goodSamplesGenes* function in WGCNA, we also explored overlap between networks by computing a cross-tabulation of the genes in 1-dph modules assigned to 1 mph modules. In other words, as module color names are specific for each data set, this analysis allows to identify—and statistically test using a Fisher exact test—how the genes contained in one module of one life stage are distributed in modules of the other life stages.

A related—but distinct—analysis across stages in the WGCNA toolkit is the computation of consensus modules. This analysis constructs modules of genes which are coexpressed across all data sets (in our case, 1 dph and 1 mph), which is clearly distinct from how well the modules constructed using a single data set are preserved in another data set. Here, we constructed a consensus network using the same parameters as we used for the analyses within stages (see above). Similarly to what we did for analyses within stages, we computed “eigengenes” and used the significance of their correlation with benthic/limnetic state (controlling for false discovery rate) to identify modules of interest. This analysis of the correlation of consensus eigengenes with benthic/limnetic state was performed separately for 1 dph and 1 mph (i.e., the modules identified are common to stages because they are consensus modules, but their eigengenes and correlation with benthic/limnetic state are computed separately for each data set). For those modules deemed significantly associated with benthic/limnetic state, we also considered as candidate transcripts those which had the absolute value of both module membership (correlation of expression with “eigengene” scores) and biweighted midcorrelation with benthic/limnetic state higher than 0.7.

### Enrichment Analysis

Enrichment analysis was performed on the gene sets included in the previously identified modules showing high association with benthic/limnetic stage (exceeding the threshold of 0.7 for both module membership and biweighted midcorrelation; see above), as well as on the genes identified as differentially expressed using DESeq2. To identify significantly over-represented Gene Ontology (GO) terms and Kyoto Encyclopedia of Genes and Genomes (KEGG) pathways in the selected genes (test sets) when compared with the whole gene set (baseline set), we used a Fisher’s exact test implemented in g:Profiler ve94_eg41_p11_9f195a1 (Reimand et al. 2007) with g:SCS multiple testing correction method applying significance threshold of 0.05 (Reimand et al. 2016). We carried out the GO enrichment tests using as baseline the closely related cichlid species Nile tilapia (*O.niloticu*s), while for the KEGG pathway analysis we used the zebrafish (*Danio rerio*) gene sets as baseline (the only fish species available in the g: Profiler database providing the KEGG analysis). 

## Results

We obtained 333.2 million (M) raw reads (from 53.4 to 84.8 M reads per species), each 146 bp in length (after removing the 5-bp barcode), a number that was reduced to 299.4 M high-quality reads (from 47.5 to 76.1 M reads per species) after the application of the filtering criteria described in the “Materials and Methods” section (supplementary table S1, Supplementary Material online). The reference-assembly procedure rendered 83,193 transcripts (*N*50 = 5,418), then reduced to 20,178 likely coding sequences (*N*50 = 6,164) after selecting the longest transcript matching the same Nile tilapia protein. The clustering approach based on multimapped reads implemented in Corset identified 17,376 clusters/genes to which 148 M reads were assigned across all the 27 samples.

### Gene Coexpression within Stages

**Fig. 2.**
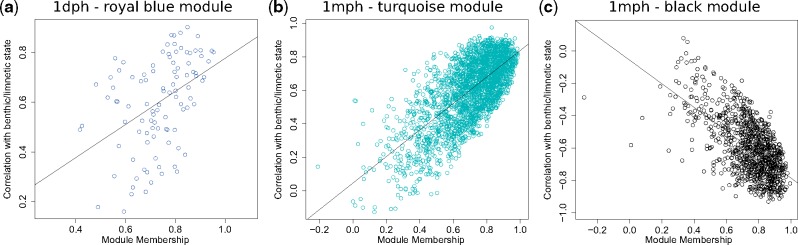
—For each transcript belonging to selected modules, the correlation with benthic/limnetic state is plotted against module membership (which is the correlation between the transcript abundance and the eigengene). A correlation is also computed between these two measures, with large and significant values indicating that transcripts which have a strong module membership also have a strong association with benthic/limnetic state. (*a*) Royal blue 1 dph; the overall correlation between benthic/limnetic state and module membership for the transcripts is 0.43 (*P* < 0.0001). (*b*) Turquoise 1 mph; the correlation between benthic/limnetic state and module membership for all the transcripts in the module is 0.71 (*P* < 0.0001). (*c*) Black 1 mph; the correlation between benthic/limnetic state and module membership for all the transcripts is −0.63 (*P* < 0.0001).

The module identification procedure based on the signed network obtained with biweight midcorrelation returned 25 modules for 1 dph (ranging from 62 to 4,006 transcripts, plus 507 transcripts not assigned to any module) and 30 for 1 mph (ranging from 39 to 2,517 transcripts, plus 104 transcripts not assigned to any module) (supplementary fig. S3, Supplementary Material online). The absolute biweight midcorrelation of “eigengenes” for each module and benthic/limnetic state ranged between 0.06 and 0.80 for 1 dph and between 0.01 and 0.84 for 1 mph. Of the 25 1 dph modules, a single module (“royal blue”) had significant correlation with benthic/limnetic state after controlling for false discovery rate (fig. 2*a*). This module contains 100 transcripts, that have positive correlation with benthic/limnetic state (i.e., they are overexpressed in limnetic species). Two of the 30 1 mph modules (“turquoise,” “black”) had significant correlation with benthic/limnetic state after controlling for false discovery rate. The “turquoise” 1 mph module is the largest of the 1 mph modules and contains 2,517 genes, whereas the “black” module contains 953 genes. While the transcripts in the “turquoise” 1 mph module have almost exclusively positive correlation with benthic/limnetic state (fig. 2*b*), the transcripts in the “black” 1 mph module have almost exclusively negative correlation with benthic/limnetic state (fig. 2*c*). Of the 100 transcripts assigned to the single 1 dph module statistically significant after controlling the false discovery rate (“royal blue”), 32 were over the threshold of 0.7 (absolute value) for both module membership (correlation of expression with “eigengene” scores) and biweighted midcorrelation with benthic/limnetic state. The genes included in this module exceeding the threshold span different functions (supplementary table S4, Supplementary Material online), but no functional categories were found to be enriched after correcting for multiple tests. For the “turquoise” and “black” modules of the 1 mph stage, 811 and 280 transcripts were over the threshold, respectively (supplementary tables S5 and S6, Supplementary Material online). In both cases, these genes, highly correlated with benthic/limnetic state, showed significant enrichment for some functional categories mainly involving GO terms associated with nervous system processes, ion transmembrane transport and receptor signaling pathways (“turquoise” module), and with protein catabolic processes (“black” module) (supplementary tables S7 and S8, Supplementary Material online). Notably, the results of the GO tests were confirmed by the KEGG analysis, where the calcium signaling and different protein processing pathways showed a significant enrichment in the “turquoise” and “black” module, respectively (supplementary tables S7 and S8, Supplementary Material online). These findings highlight the potentially important role of these cellular functions for the benthic/limnetic axis of divergence.

All modules selected with the procedure based on biweighted midcorrelation with benthic–limnetic state and accounting for false discovery rate had a significantly large explained variance of the first component (“eigengene,” in all cases *P* < 0.001). Similarly, all these modules had a significant multivariate association with benthic/limnetic state, as tested with the permutation procedure based on the Escoufier RV coefficient (*P* = 0.02 for the “black” 1 mph module, *P* < 0.01 for the other two modules).

### Analyses of Differential Gene Expression

Our analyses of gene expression for the 1 dph stage revealed 53 genes differentially expressed between benthic and limnetic fish after controlling for false discovery rate (supplementary table S9, Supplementary Material online). Of these, four were in the “royal blue” selected module. The same analysis returned a total of 305 differentially expressed genes when performed on the 1 mph stage (supplementary table S10, Supplementary Material online). Of these, 57 belonged to the “turquoise” and 67 to the “black” module. In all cases, the selected modules were significantly enriched in differentially expressed genes (Fisher exact test, *P* value ranging from 0.00024 of the “royal blue” 1 dph module to 7.9e^−25^ of the “black” 1 mph module).

The 53 differentially expressed genes of the 1 dph stage are significantly enriched for the GO term “proteasome core complex” (supplementary table S11, Supplementary Material online). Conversely, the genes deemed differentially expressed between benthic and limnetic fish at 1 mph are significantly enriched for 50 GO terms, spanning molecular function, biological process and cellular component, and five KEGG pathways (supplementary table S12, Supplementary Material online).

### Overlap of Selected Modules and Previously Identified QTL

The level of overlap between genes in the modules deemed as significantly associated with benthic/limnetic state and previously identified QTL regions greatly varied (Additional file 5: Table S8) from no transcript in the QTL region (i.e., royal blue 1 dph and “QTL for covariation” of body and pharyngeal jaw shape) to 255 transcripts in QTL regions (i.e., turquoise 1 mph and all QTL regions combined). A large variability in the number of overlaps as function of the size of the QTL regions as well as the number of genes in a module is expected and motivates the need for hypothesis testing. Of all the tests we performed, only two were statistically significant (transcripts in the turquoise 1 mph module with, respectively, the QTL regions for pharyngeal jaw shape and “covariation” between body and pharyngeal jaw shape). However, neither of these was significant after controlling for false discovery rate.

A similar picture emerged when analyzing the overlap of differentially expressed genes and QTL regions. These ranged from zero overlaps (differentially expressed 1 dph genes in target modules vs QTL for pharyngeal jaw shape; genes both differentially expressed and in target modules vs QTL for covariation) to 38 (differentially expressed 1 mph genes and all QTL regions combined). However, in no case this overlap was significant using the permutation procedure implemented in *regioneR*.

### Exploratory Analyses of Global Transcription Profiles

**Fig. 3.**
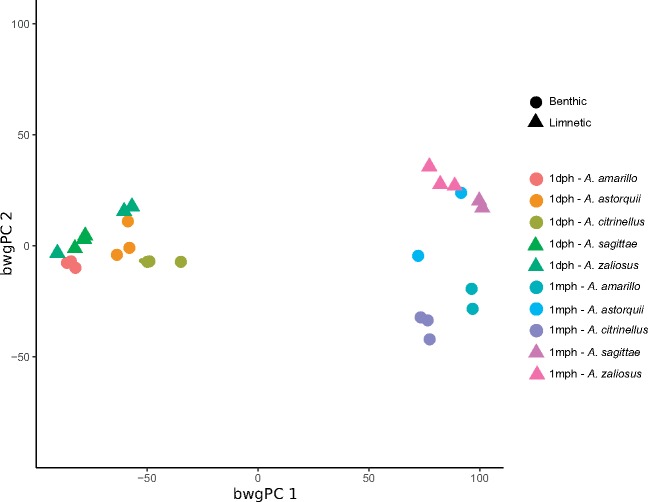
—Scores along the first two between-group principal components (bwgPC) obtained using each individual combination of stage (1 dph and 1 mph) and morph (benthic and limnetic) as factor.

The computation of Euclidean distances between benthic and limnetic fish across stages reveals that at 1 dph benthic and limnetic fish are transcriptionally more similar (Euclidean distance 27.5) than at 1 mph (Euclidean distance 45.3). The exploratory plot (fig. 3) of the scores along the first two between-group principal components (which together account for 80.16% of the original variation among individual observations) confirms this and shows that gene expression variation among life stages is larger than variation between benthic and limnetic fish. This plot also shows clearly diverging “allometric trajectories” between benthic and limnetic fish, that are much more distinct at 1 mph than at 1 dph. The plot further suggests that at 1 mph differences among species and lakes are more pronounced than at 1 dph. Interestingly, a plot of the scores on the third between-group principal component (supplementary fig. S13, Supplementary Material online) shows some level of separation between benthic and limnetic fish at 1 dph, but this is less pronounced than the one observed at 1 mph (fig. 3). The scatterplot of the scores along the first two between-group principal components also shows some level of overlap between benthic and limnetic fish at 1 mph. This is not surprising, as between-group principal component analysis is an exploratory technique aimed at providing a low-dimensional representation of differences between groups (Mitteroecker and Bookstein 2011).

### Comparison of Modules across Stages

**Fig. 4.**
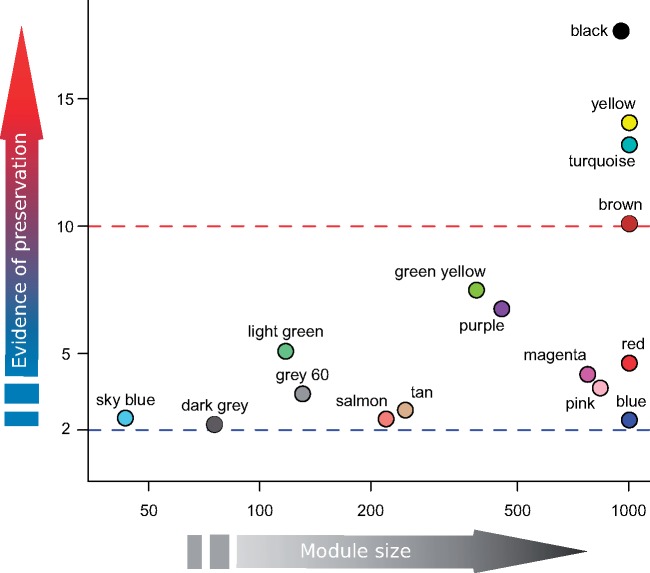
—Plot of module preservation versus module size. Module preservation is expressed using the *Z*_summary_ statistic, module size is the number of transcripts belonging to a given module. The *Z*_summary_ statistic quantifies the level of preservation of the modules of the 1 mph coexpression network in the 1 dph coexpression network, therefore, the color names refer to the colors of the 1 mph network (supplementary figure S3, Supplementary Material online). The blue line indicates the threshold (*Z*_summary_=2) below which there is no evidence of preservation, whereas the red line indicates the threshold (*Z*_summary_=10) above which there is strong evidence of preservation. The modules with *Z*_summary_<2 have been removed from this figure, but a complete version of the plot can be found in supplementary figure S14, Supplementary Material online.

Our analysis of module preservation (fig. 4; supplementary fig. S14, Supplementary Material online) revealed strong evidence of preservation for four 1 mph modules in the 1 dph module set. These four modules, identified as their *Z*_summary_ statistic was higher than 10 (Langfelder et al. 2011), are—in decreasing order of preservation—the “black,” “yellow,” “turquoise,” and “brown” modules. It is worth noticing that the “black” and “turquoise” modules of the 1 mph coexpression network are significantly associated with benthic/limnetic state (see above) and that the included genes showed enrichment in several functional categories, mainly related with nervous system, ion transmembrane transport, receptor signaling, and protein catabolic processes (supplementary tables S7 and S8, Supplementary Material online). The “turquoise” module is also the largest of the 1 mph modules. The analysis also identified 14 modules without evidence of preservation (*Z*_summary_ lower than 2) and 14 modules with some evidence of preservation (intermediate values of *Z*_summary_). The overlap table (supplementary table S15, Supplementary Material online) reveals that the only 1 dph module robustly associated with benthic/limnetic state (i.e., “royal blue”) significantly overlaps with the “turquoise” 1 mph module (28 genes), as well as two other 1 mph modules. Remarkably, of the 28 genes overlapping between the “royal blue” 1 dph module and the “turquoise” 1 mph module, 22 were among those selected in one or both of the within-stage analyses.

The computation of consensus modules identified 58 modules (i.e., approximately double than the number of the modules obtained separately within each life stage), containing between 46 and 1,552 transcripts, with an impressive 1,162 transcript not classified in any module. We found another interesting result when testing for the association between consensus modules and benthic/limnetic state. This analysis, performed using the genes in the consensus modules but testing their association separately for the 1 dph and 1 mph stage, allowed us to identify significant associations after controlling for false discovery rate only at 1 mph. In particular, this is the case for three consensus modules. In other words, while these modules represent genes coexpressing in both life stages, their association with benthic/limnetic state is stronger/more easily identified at the 1 mph. These three modules, in turn, contain 287 (“red” consensus module), 73 (“light green” consensus module), and 58 (“dark turquoise” consensus module) transcripts robustly assigned to module and associated with benthic/limnetic state (supplementary tables S16–S18, Supplementary Material online). These three modules include genes involved in different biological functions, but only the “red” consensus module showed significant enrichment of GO and KEGG terms associated processes that mirrored those found in the 1 mph “turquoise” module identified in the gene coexpression analysis within stages (see above) (supplementary table S19, Supplementary Material online).

## Discussion

By conducting an analysis of gene (co)expression at two life stages of five extremely young Midas cichlid fish species, we identified two main patterns: 1) a substantial variation between the two life stages; 2) higher divergence between benthic and limnetic fish species later in development. The reduced sample sizes (number of individuals) requires caution in interpreting the results. However, we have taken many steps to minimize the chances of false positives and the patterns are so clear that we are confident that future studies based on larger sample sizes will confirm and refine these findings.

In detail, the variation in patterns of gene (co)expression between life stages appears much larger and more evident than the variation between benthic and limnetic species, individual species and lakes of origin. This is perhaps unsurprising considering how different morphologically, developmentally and ecologically are the two life stages we have considered in this study, and how comparatively similar—and recently diverged—are benthic and limnetic fish. In fact, only very recently seven small and young crater lakes (Lake Apoyo, the oldest Nicaraguan crater lakes, is maximally approximately, 22,000 year old; Kutterolf et al. 2007; Kautt et al. 2016) were colonized independently from the great lakes Managua and Nicaragua (>500,000 years old), that are inhabited by benthic species. Within two of these crater lakes, Apoyo and Xiloá, the colonizers have undergone sympatric speciation, and independently evolved similar phenotypes in each lake (Barluenga et al. 2006; Elmer et al. 2014) producing a few benthic species and one limnetic species (Elmer et al. 2014). Because of the limited independent evolutionary time (great lakes vs crater lake species) and the still incomplete reproductive isolation process (sympatric crater lake species), genetic divergence within the Midas species complex is relatively low (e.g., *F*_ST_ = 0–0.08; Elmer et al. 2014; Kautt et al. 2016).

One might have expected that the entirely different needs of being a larva absorbing the yolk sac and being a fully formed free-swimming juvenile fish are much larger than the differences between recently diverged open-water and bottom-dwelling benthos-associated fish. Nonetheless, it seems remarkable to find such large and (almost) all-encompassing changes in patterns of coexpression.

We do document that some groups of coexpressing genes (modules) are preserved across life stages. In particular, we find strong evidence for four large 1 mph modules preserved in 1 dph fish and various other modules with some evidence of preservation. Notably, two of these four modules showed strong association with the benthic/limnetic state. GO and KEEG analysis identified significant enrichment of several terms associated with nervous system processes, ion transmembrane transport, receptor signaling and with protein catabolic processes. This finding might suggest that these biological pathways could play an important role in promoting the benthic/limnetic axis of divergence. However, the main expression pattern is one of discordance between life stages, not only with different genes being expressed at different stages (e.g., as shown in the plot of the scores along the first two between-group principal components: fig. 3) but also, substantially, rearranging in new covariation blocks. This is particularly evident when comparing the makeup of the consensus modules to modules constructed separately for each life stage: Starting with the same genes, consensus modules are many more and smaller than either of the two stage-specific modules. As consensus modules are groups of genes showing consistent patterns of coexpression across life stages, their “parcellation” compared with either stage reveals that much smaller groups of genes are indeed consistently coexpressing across life stages. Most remarkably, many genes cannot be assigned to any consensus module, further revealing a lack of consistency in patterns of coexpression across life stages. What we observe in the Midas cichlid system is in line with research that aimed at understanding the modularity of gene expression during ontogeny (Raff and Sly 2000; Lorenz et al. 2011; Jimenez et al. 2017). Animals consist of hierarchically organized structural and functional subunits, a modular organization that is not static during development (Raff 1996). These different and dynamic developmental modules have a discrete organization defined by the expression of specific sets of genes, and thus pattern of coexpressed genes (modules) expected to vary during animal development (Raff and Sly 2000).

Turning to patterns of divergence between benthic and limnetic fish, our results clearly support the hypothesis of increased divergence in the later life stage, with clearly diverging “allometric trajectories.” This is particularly clear observing the plot of the scores along the first two between-group principal components of figure 3. The plot can be thought as a representation of the overall patterns of (co)expression in our samples and shows that the transcriptomes of benthic and limnetic species are more distinct at the free-swimming 1 mph stage than at the larva 1 dph stage. Interestingly, also variation among species and among lakes appear larger at the 1 mph stage. We do find genes differentially expressed between benthic and limnetic species at 1 dph and we even document an entire, fairly small, module of coexpressing genes robustly associated with benthic/limnetic state in this life stage. Even though no functional categories showed significant enrichment, these genes may be associated with physiological differences between benthic and limnetic fish which develop earlier. However, at 1 mph we find two large modules (in fact one of the two is the largest module found at this life stage) associated with benthic/limnetic state, one of them with genes coexpressing and consistently upregulated in limnetic fish, the other with genes coexpressing and consistently upregulated in benthic fish. In other words, a much larger number of genes—and of groups of coexpressing genes—appears to be associated with the benthic–limnetic axis of divergence at 1 mph.

Clearly, we expect that the observed patterns of strong divergence between life stages and the increase in benthic/limnetic divergence with age should “plateau” with growth, perhaps becoming even parallel with adulthood. However, this is currently merely speculation and future studies should further address the ontogenetic component of variation in (co)expression. In the context of the knowledge of the biology of these fish it is already remarkable, however, that substantial differences in gene (co)expression are found at such young age. It should be noticed, indeed, that while at 1 mph Midas cichlids are free swimming, it is doubtful whether they are already divergent in actual swimming and feeding habits at such a young age. If these fish do not exhibit adult swimming and feeding habits at 1 mph, then, the divergent swimming and feeding habits in adults have evolved in the presence of gene flow through a large amount of changes in expression effected at multiple life stages.

For further understanding sympatric speciation using Midas cichlids as a model, another result is worth discussing: The lack of clear overlap between genes in modules associated with benthic/limnetic ecology and recently identified QTL regions for body and pharyngeal jaw morphology (Fruciano et al. 2016a). It should be noticed that we do find two cases in which we observe significant overlap of a module and a QTL, but these do not hold after controlling for false discovery rate and must be, at this stage, considered as “false positives.” There are various, nonmutually exclusive, explanations to the lack of significant overlap between gene coexpression modules and QTL regions. First of all, it is well known that QTL mapping studies have low detection power (Beavis 1998; Schon et al. 2004), thus it is fair to assume that variation in body and pharyngeal jaw shape between benthic and limnetic fish is due to a larger number of QTL regions than we have found in our earlier genetic mapping studies (Franchini et al. 2014b; Fruciano et al. 2016a) (i.e., there are many more genes of smaller effect contributing to the variation in body and pharyngeal jaw shape). Most likely, more QTL regions would mean higher levels of overlap with the genes in our selected modules. Furthermore, QTL regions for the same traits in the two lakes where distinct benthic/limnetic forms have evolved (crater lakes Apoyo and Xiloá) do not necessarily have to be the same (or have the same effect sizes) (Schielzeth et al. 2018). We have previously identified QTL regions only in fish from Lake Apoyo (QTL mapping on fish from Lake Xiloá is currently underway) whereas in this study we have used fish from both crater lakes (and from the source Lake Nicaragua). The genetic architecture of these traits could be different in the Apoyo and Xiloá radiations (i.e., different genes might be used to produce a similar phenotypic divergence, perhaps with differential use of ancestral polymorphism), and this would lead to further underestimating the level of overlap between QTL regions and genes in selected modules. Also, it is entirely possible that the overlaps we currently consider false positives are, indeed, true positives. Clearly, future studies with larger sample sizes will have higher power in detecting overlaps with QTL regions by virtue of further refinement of selected modules. Another plausible explanation could be found in our experimental design. On one end, the use of whole organism-derived expression data allowed us to capture general patterns of gene expression and coexpression. On the other hand, this approach could prevent the identification of overlaps between gene expression and QTL in case this latter controls the variation of traits by targeting specific tissues/organs during development. Finally, the idea that one should find significant overlap between QTL regions and modules of coexpressed genes significantly associated with benthic/limnetic divergence hinges on the assumption that the vast majority of gene expression regulation is due to *cis* regulatory elements (here cis defined to be “close enough that both gene and regulatory element could be in the same QTL region”).

In any case, by explicitly tackling the nonindependence among expressed genes, here we have identified divergent “allometric trajectories” in gene expression between sympatrically diverged cichlids and identified a set of modules of coexpressing genes associated with benthic–limnetic divergence.

## Conclusions

Our results showed that divergence over development between benthic–limnetic species is accompanied by divergence in patterns of (co)expression of several genes. In detail, we observed that later in the development more and larger modules of coexpressing genes differentiate the two morphs. Our analyses suggest that what might be most critical during adaptive divergence is a complex and extensive post-transcriptional regulation that drives the (co)expression of a large number of genes.

## Supplementary Material

Supplementary data are available at *Genome Biology and Evolution* online. 

## Supplementary Material

Supplementary_Material_evz108

## Notes

**Data deposition:** Raw Illumina sequences have been deposited into the NCBI’s Sequence Read Archive (SRA) database with accession number PRJNA544930.
