# Supplementary material for: Divergent Allometric Trajectories in Gene Expression and Coexpression Produce Species Differences in Sympatrically Speciating Midas Cichlid Fish
Source: Genome Biol Evol. 2019 May 24;11(6):1644–57. doi: 10.1093/gbe/evz108 (PMC6563553; doi:10.1093/gbe/evz108)
Supplement: Supplementary_Material_evz108 [file supplementary_material_evz108.zip › suppl legends.docx]

**Supplementary material legends**

**Supplementary Table S1.** Description and sequencing statistics of the 39 samples used in this study. Library ID, species, lake of origin, phenotype, developmental stage and number or reads before and after filtering are shown. The last column describes the final samples used in the statistical analyses.

**Supplementary Figure S2.** Flowchart describing the main analyses related to WGCNA (Weighted Correlation Network Analysis) performed in this study. Orange boxes indicate input files (i,e, gene expression data). Blue boxes indicate downstream analyses.

**Supplementary Figure S3.** Signed gene co-expression networks based on biweight midcorrelation for each growth stage. Each dendrogram clusters transcripts based on their biweight midcorrelation. In the first band, each colour represents a different module identified by the procedure. Notice that corresponding colours across the two networks do not indicate the same modules. In the second band, the strength of the biweight midcorrelation between each transcript expression and a binary value representing benthic/limnetic state (0=benthic, 1=limnetic) is shown. Red lines represent positive correlation (i.e., transcript over-expressed in the limnetic species relative to the benthic species) while blue lines indicate negative correlation.

**Supplementary Table S4.** Description, correlation with phenotype, correlation with module membership and annotation of the 32 genes highly correlated with the benthic/limnetic state included in the 1 dph “royal blue” module.

**Supplementary Table S5.** Description, correlation with phenotype, correlation with module membership and annotation of the 811 genes highly correlated with the benthic/limnetic state included in the 1 mph “turquoise” module.

**Supplementary Table S6.** Description, correlation with phenotype, correlation with module membership and annotation of the 280 genes highly correlated with the benthic/limnetic state included in the 1 mph “black” module.

**Supplementary Table S7.** Significantly enriched Gene Ontology (GO) categories and KEGG pathways for the 811 genes included in the 1 mph “turquoise” module (described in Supplementary Table S5). The genes belonging to each GO and KEGG term are reported in the last column with their abbreviated name.

**Supplementary Table S8.** Significantly enriched Gene Ontology (GO) categories and KEGG pathways for the 280 genes included in the 1 mph "black" module (described in Supplementary Table S6). The genes belonging to each GO and KEGG term are reported in the last column with their abbreviated name.

**Supplementary Table S9.** Description, expression difference (fold change), statistical significance and annotation of the 53 differentially expressed (DE) genes between benthic and limnetic fishes at 1 dph. Positive log2(fold change) values indicate higher expression in limnetic fishes.

**Supplementary Table S10.** Description, expression difference (fold change), statistical significance and annotation of the 305 differentially expressed (DE) genes between benthic and limnetic fishes at 1 mph. Positive log2(fold change) values indicate higher expression in limnetic fishes.

**Supplementary Table S11.** Significantly enriched Gene Ontology (GO) categories for the 53 differentially expressed (DE) genes between benthic and limnetic fishes at 1 dph (described in Supplementary Table S9). The genes belonging to each GO term are reported in the last column with their abbreviated name.

**Supplementary Table S12.** Significantly enriched Gene Ontology (GO) categories and KEGG pathways for the 305 differentially expressed (DE) genes between benthic and limnetic fishes at 1 mph (described in Supplementary Table S10). The genes belonging to each GO and KEGG term are reported in the last column with their abbreviated name.

**Supplementary Figure S13.** Scores along the first and the third between-group principal components (bwgPC) obtained using each individual combination of stage (1 dph and 1 mph) and morph (benthic and limnetic) as factor.

**Supplementary Figure S14.** Plot of the Z_summary_ statistic versus module size (number of transcripts). Here, this statistic is used to quantify the level of preservation of the modules of the 1 mph co-expression network in the 1 dph co-expression network, therefore, the color names refer to the colors of the 1 mph network. Larger values of the Z_summary_ statistic imply a stronger evidence of preservation of the module – as identified in the 1 mph network – in the 1 dph network. The blue line indicates the threshold (Z_summary_=2) below which there is no evidence of preservation, whereas the red line indicates the threshold (Z_summary_=10) above which there is strong evidence of preservation.

**Supplementary Table S15.** Overlap of modules across life stages. The first table (Table S15a) reports the number of genes of each 1 dph module (rows) assigned to each 1 mph module (columns), the second (Table S15b) the p-values of the Fisher exact test. Highlighted are the only 1 dph module robustly identified as associated to the benthic/limnetic state (“royal blue”) and, using borders, the four 1 mph modules with strong evidence for preservation in the 1 dph module (i.e., “black”, “turquoise”, “yellow”, and “brown”). Significant overlaps between the “royal blue” 1 dph genes and 1 mph modules are in bold. The third table (Table S15c) focuses specifically on the 28 genes overlapping between the “royal blue” 1 dph and the “turquoise” 1 mph modules and reports whether these were among the selected genes for the analyses within life stages (values for both correlation with benthic/limnetic state and module membership higher than 0.7).

**Supplementary Table S16.** Description, correlation with phenotype, correlation with module membership and annotation of the 287 genes highly correlated with the benthic/limnetic state included in the consensus “red” module.

**Supplementary Table S17.** Description, correlation with phenotype, correlation with module membership and annotation of the 58 genes highly correlated with the benthic/limnetic state included in the consensus “dark turquoise” module.

**Supplementary Table S18.** Description, correlation with phenotype, correlation with module membership and annotation of the 73 genes highly correlated with the benthic/limnetic state included in the consensus “light green” module.

**Supplementary Table S19.** Significantly enriched Gene Ontology (GO) categories and KEGG pathways for the 287 genes highly correlated with the benthic/limnetic state included in the consensus “red” module (described in Supplementary Table S16). The genes belonging to each GO and KEGG term are reported in the last column with their abbreviated name.
