# Supplementary figures and images for: Divergent Allometric Trajectories in Gene Expression and Coexpression Produce Species Differences in Sympatrically Speciating Midas Cichlid Fish
Source: Genome Biol Evol. 2019 May 24;11(6):1644–57. doi: 10.1093/gbe/evz108 (PMC6563553; doi:10.1093/gbe/evz108)

# WGCNA workflow

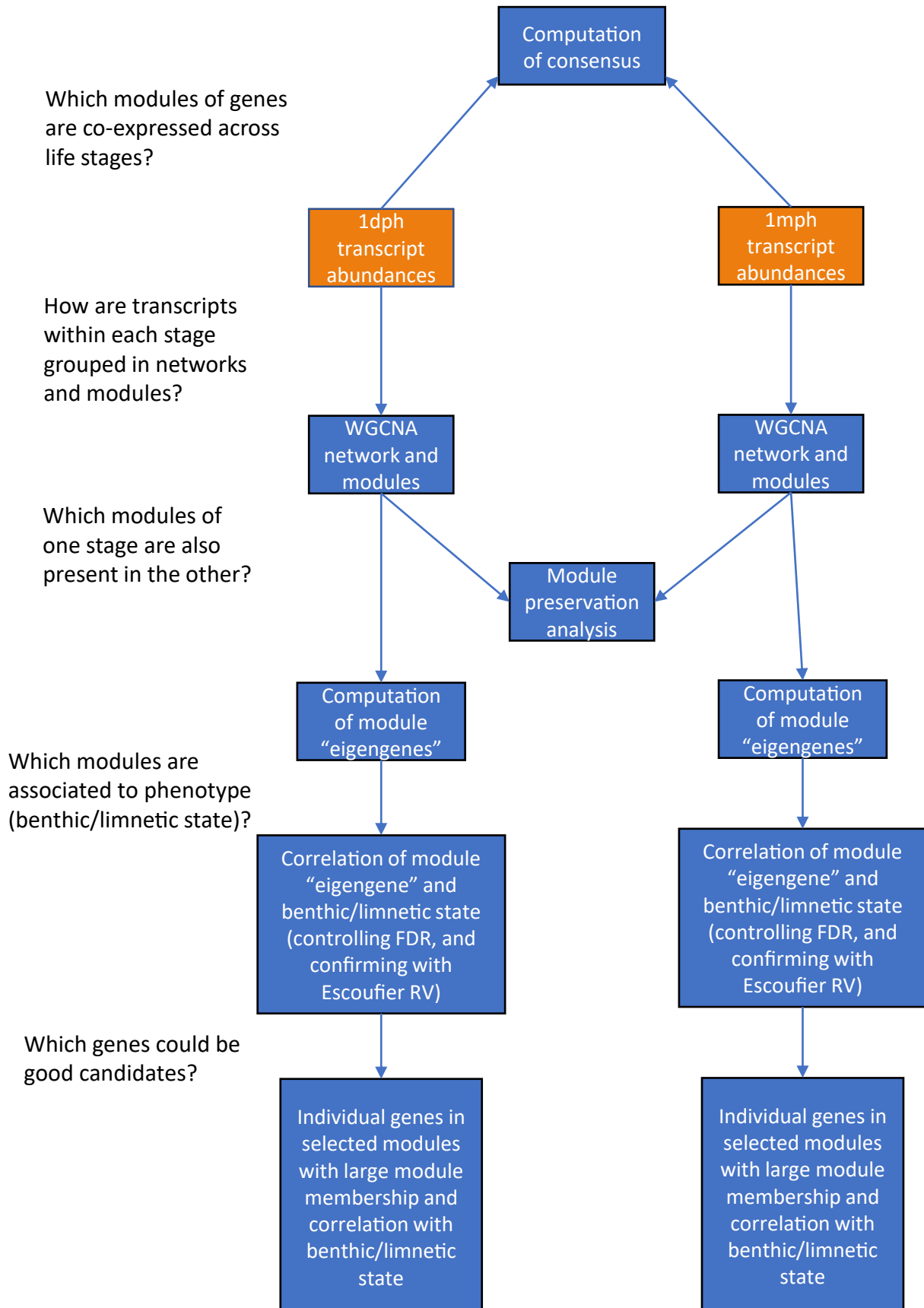

Supplement: Supplementary_Material_evz108 [file supplementary_material_evz108.zip › 02.Supplementary_Fig_S2_REV2.pdf]

1 dph

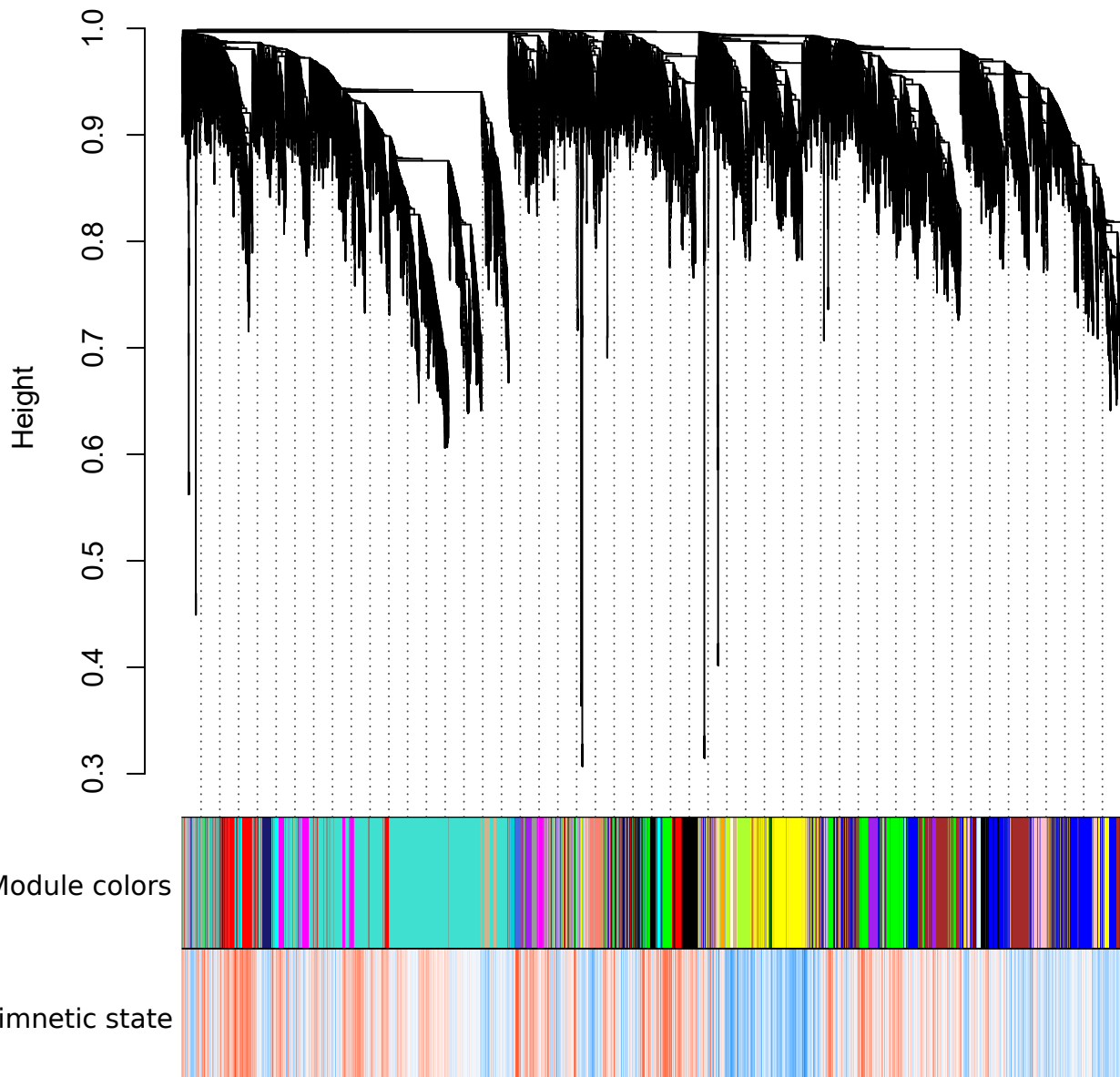

1 mph

Height

1.00  
0.95  
0.90  
0.85  
0.80  
0.75  
0.70  
0.65

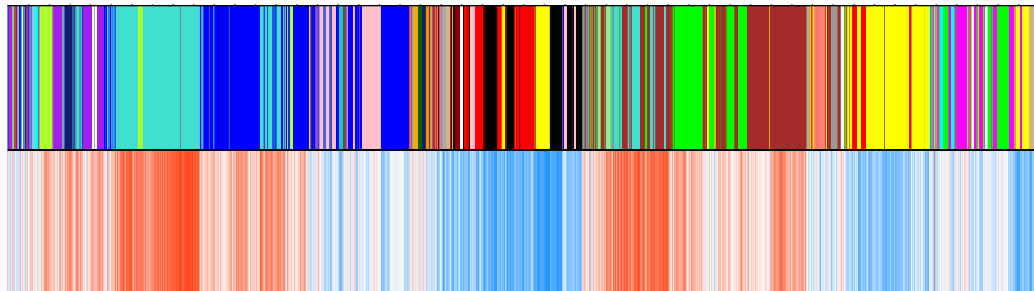

Supplement: Supplementary_Material_evz108 [file supplementary_material_evz108.zip › 03.Supplementary_Fig_S3_REV2.pdf]

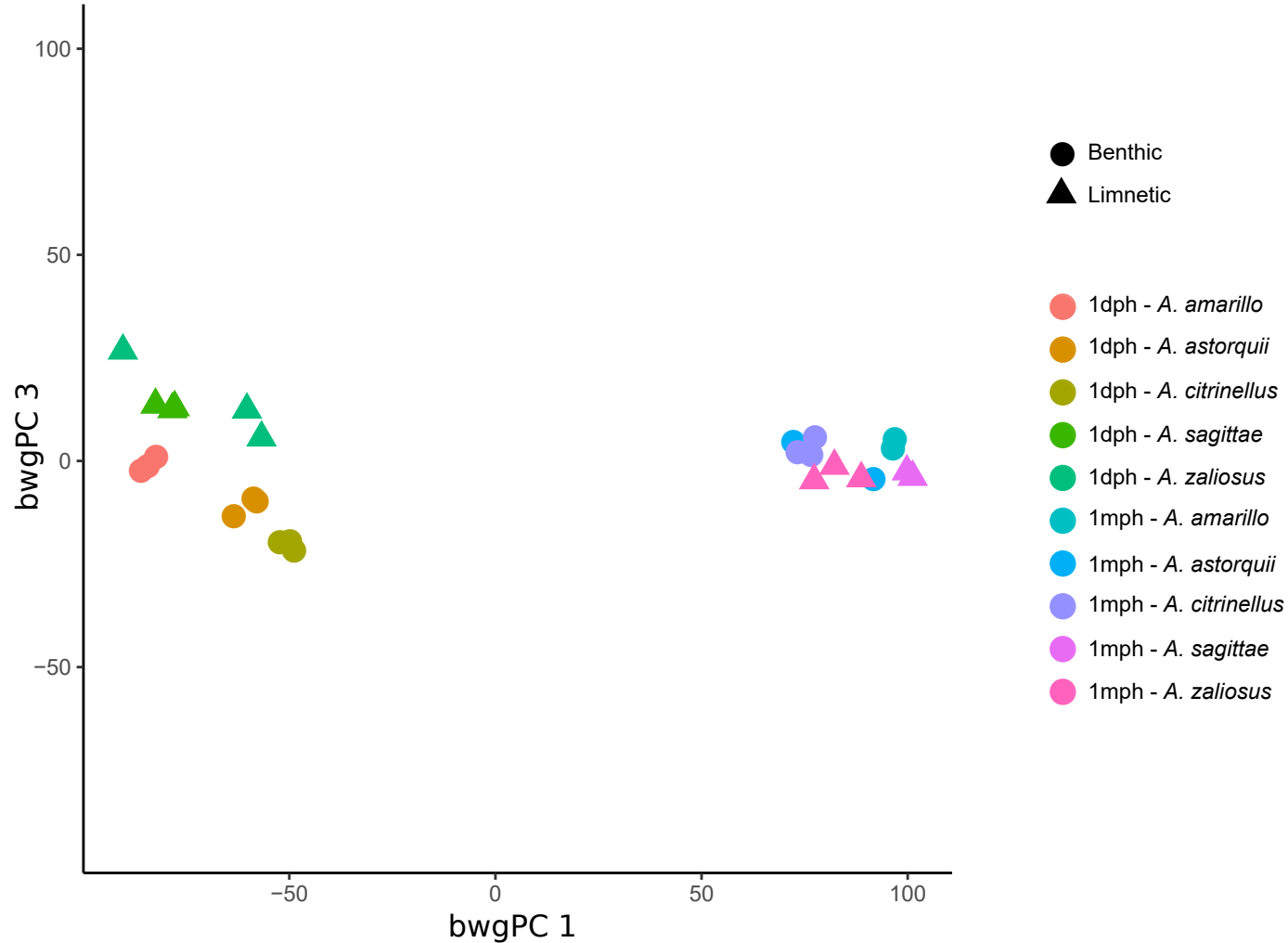

Supplement: Supplementary_Material_evz108 [file supplementary_material_evz108.zip › 08.Supplementary_Fig_S13_REV2.pdf]

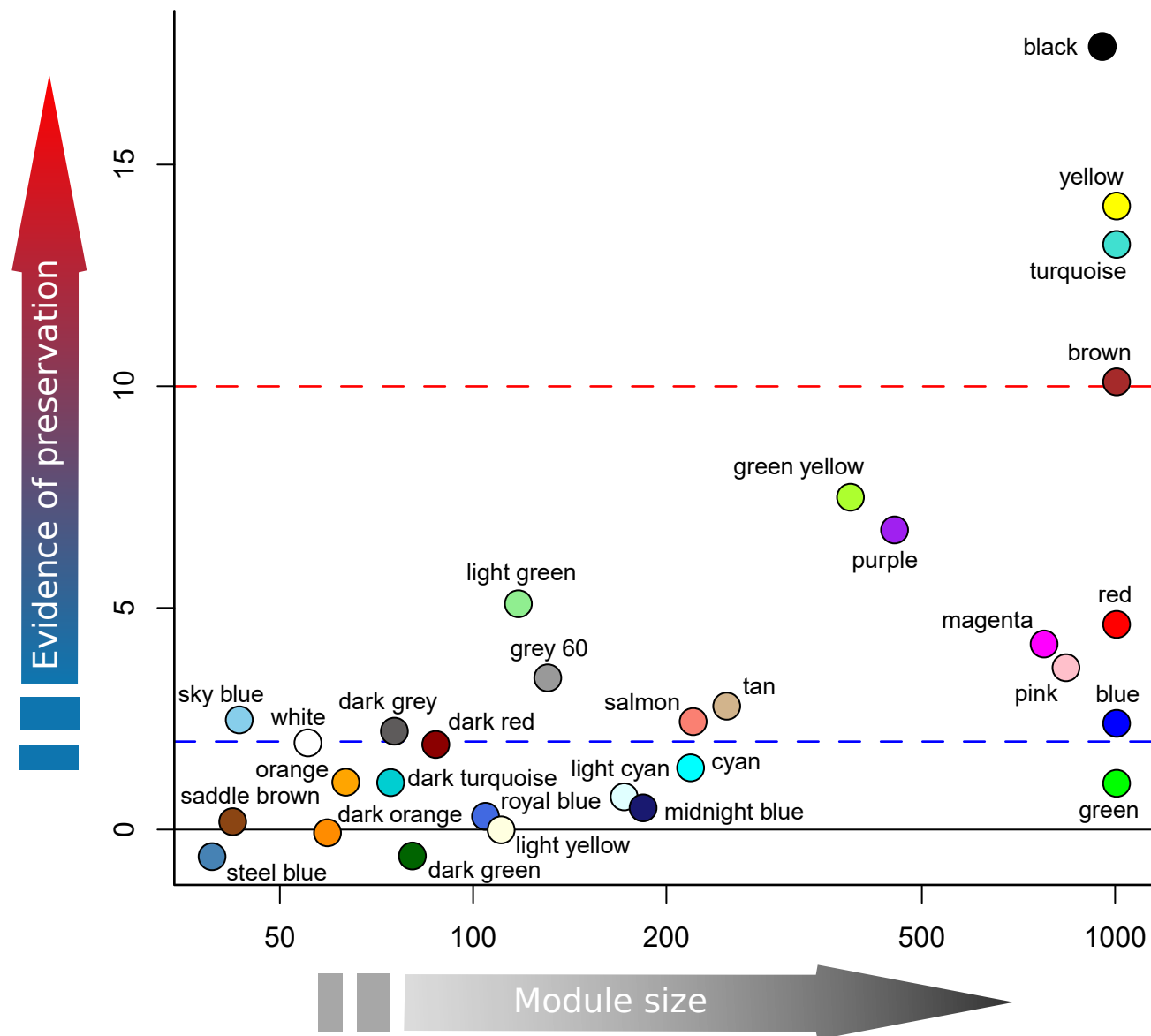

Supplement: Supplementary_Material_evz108 [file supplementary_material_evz108.zip › 09.Supplementary_Fig_S14_REV2.pdf]
